# Development and validation of a three-dimensional deep learning-based system for assessing bowel preparation on colonoscopy video

**DOI:** 10.3389/fmed.2023.1296249

**Published:** 2023-12-18

**Authors:** Lina Feng, Jiaxin Xu, Xuantao Ji, Liping Chen, Shuai Xing, Bo Liu, Jian Han, Kai Zhao, Junqi Li, Suhong Xia, Jialun Guan, Chenyu Yan, Qiaoyun Tong, Hui Long, Juanli Zhang, Ruihong Chen, Dean Tian, Xiaoping Luo, Fang Xiao, Jiazhi Liao

**Affiliations:** ^1^Department of Gastroenterology, Tongji Hospital of Tongji Medical College, Huazhong University of Science and Technology, Wuhan, China; ^2^Wuhan United Imaging Healthcare Surgical Technology Co., Ltd., Wuhan, China; ^3^Changzhou United Imaging Healthcare Surgical Technology Co., Ltd., Changzhou, China; ^4^Suzhou Institute of Biomedical Engineering and Technology, Chinese Academy of Sciences, Suzhou, China; ^5^Department of Gastroenterology, Yichang Central People’s Hospital, China Three Gorges University, Yichang, China; ^6^Department of Gastroenterology, Tianyou Hospital, Wuhan University of Science and Technology, Wuhan, China; ^7^Department of Gastroenterology, Hubei Provincial Hospital of Traditional Chinese Medicine, Wuhan, China; ^8^Department of Gastroenterology, Affiliated Hospital of Hubei University of Chinese Medicine, Wuhan, China; ^9^Department of Gastroenterology, Xiantao First People’s Hospital Affiliated to Yangtze University, Wuhan, China; ^10^Department of Pediatrics, Tongji Hospital of Tongji Medical College, Huazhong University of Science and Technology, Wuhan, China

**Keywords:** colonoscopy, bowel preparation, artificial intelligence, deep learning, convolutional neural network (CNN)

## Abstract

**Background:**

The performance of existing image-based training models in evaluating bowel preparation on colonoscopy videos was relatively low, and only a few models used external data to prove their generalization. Therefore, this study attempted to develop a more precise and stable AI system for assessing bowel preparation of colonoscopy video.

**Methods:**

We proposed a system named ViENDO to assess the bowel preparation quality, including two CNNs. First, Information-Net was used to identify and filter out colonoscopy video frames unsuitable for Boston bowel preparation scale (BBPS) scoring. Second, BBPS-Net was trained and tested with 5,566 suitable short video clips through three-dimensional (3D) convolutional neural network (CNN) technology to detect BBPS-based insufficient bowel preparation. Then, ViENDO was applied to complete withdrawal colonoscopy videos from multiple centers to predict BBPS segment scores in clinical settings. We also conducted a human-machine contest to compare its performance with endoscopists.

**Results:**

In video clips, BBPS-Net for determining inadequate bowel preparation generated an area under the curve of up to 0.98 and accuracy of 95.2%. When applied to full-length withdrawal colonoscopy videos, ViENDO assessed bowel cleanliness with an accuracy of 93.8% in the internal test set and 91.7% in the external dataset. The human-machine contest demonstrated that the accuracy of ViENDO was slightly superior compared to most endoscopists, though no statistical significance was found.

**Conclusion:**

The 3D-CNN-based AI model showed good performance in evaluating full-length bowel preparation on colonoscopy video. It has the potential as a substitute for endoscopists to provide BBPS-based assessments during daily clinical practice.

## Introduction

Colonoscopy is an important approach for diagnosing, monitoring, and treating colorectal diseases (1–3). As is well known, bowel preparation quality determines the visualization area of the colonic mucosa, which is a prerequisite for effective examination of various diseases, such as polyps, cancer, inflammatory bowel disease (IBD), ischemic colitis, radiation colitis, and others (4, 5). Unsatisfactory bowel preparation is closely associated with a reduced adenoma detection rate (5–7) and an increased cecal intubation failure rate (8). In addition, individuals with inadequate bowel preparation should be recommended to repeat the examination within 1 year (9, 10), which imposes a heavy burden on the medical system (11).

Several bowel preparation scales are extensively validated, including the Boston bowel preparation scale (BBPS) (12, 13), the Aronchick scale (14), and the Ottawa Bowel preparation scale (15). The BBPS is widely accepted as the best option (16, 17). According to BBPS, the colon should be divided into three segments (i.e., right, transverse, and left colon) and evaluated for bowel cleanliness. However, there are subjective differences in the understanding of BBPS among different endoscopists, and even one endoscopist may provide inconsistent evaluations at different times. In addition, the BBPS is often evaluated from one’s memory after completing the colonoscopy, which may introduce inaccuracies. Moreover, due to fatigue or time constraints, not all endoscopists are willing to record the bowel preparation quality. It was found that only 40%–62% of the reports documented the quality of bowel preparation (18–20). Therefore, an objective, reproductive, and automatic BBPS scoring system is needed in clinical practice.

In recent years, the explosive development of artificial intelligence (AI) has rapidly penetrated many fields, including medicine (21). Deep learning technology, a subset of AI, has been successfully used to classify and diagnose various clinical entities and demonstrated comparable performance to experienced clinicians (22). Several studies have assessed bowel preparation quality using AI systems trained with endoscopic images (23–25). The accuracy of these AI models applied to images can reach 93.3%–95.3% but decrease to 88.6%–89.0% in real colonoscopy video evaluation (23, 24). Another image-based model developed by Lee had 85.3% accuracy in discriminating inadequate bowel preparation in the validation set of 10 s videos (25). One possible reason for the relatively low accuracy of video evaluation may be due to the video data samples not being learned by the system. Besides, only one study tested the performance of the model on external sets (24).

The commonly used bowel preparation scoring methods during clinical practice are based on the condition of three segments of the colon or the entire colon. Therefore, evaluating images cannot reflect real clinical scenarios. It is necessary to improve the performance of AI models for evaluating bowel preparation in videos. It has been pointed out that when recognizing human action, enabling AI to learn videos directly in the training phase through a three-dimensional (3D) convolutional neural network (CNN) technology is beneficial to improve AI performance and robustness (26). Notably, video learning and 3D-CNN technology have become a trend in many areas to increase AI performance (27–29), including polyp detection (30, 31). However, no research has been published on 3D-CNN for bowel preparation assessment.

In our study, we developed a novel AI system named ViENDO to “view the endoscopy videos” and rate the degree of bowel preparation. Our approach differs from previous ones because ViENDO was directly trained with real colonoscopy videos through 3D-CNN technology instead of endoscopic images. To verify the performance in real clinical settings, we tested ViENDO’s ability to precisely score bowel cleanliness using full-length withdrawal colonoscopy videos from multiple centers and competed with endoscopists.

## Method

### Data preparation

**Figure 1:**
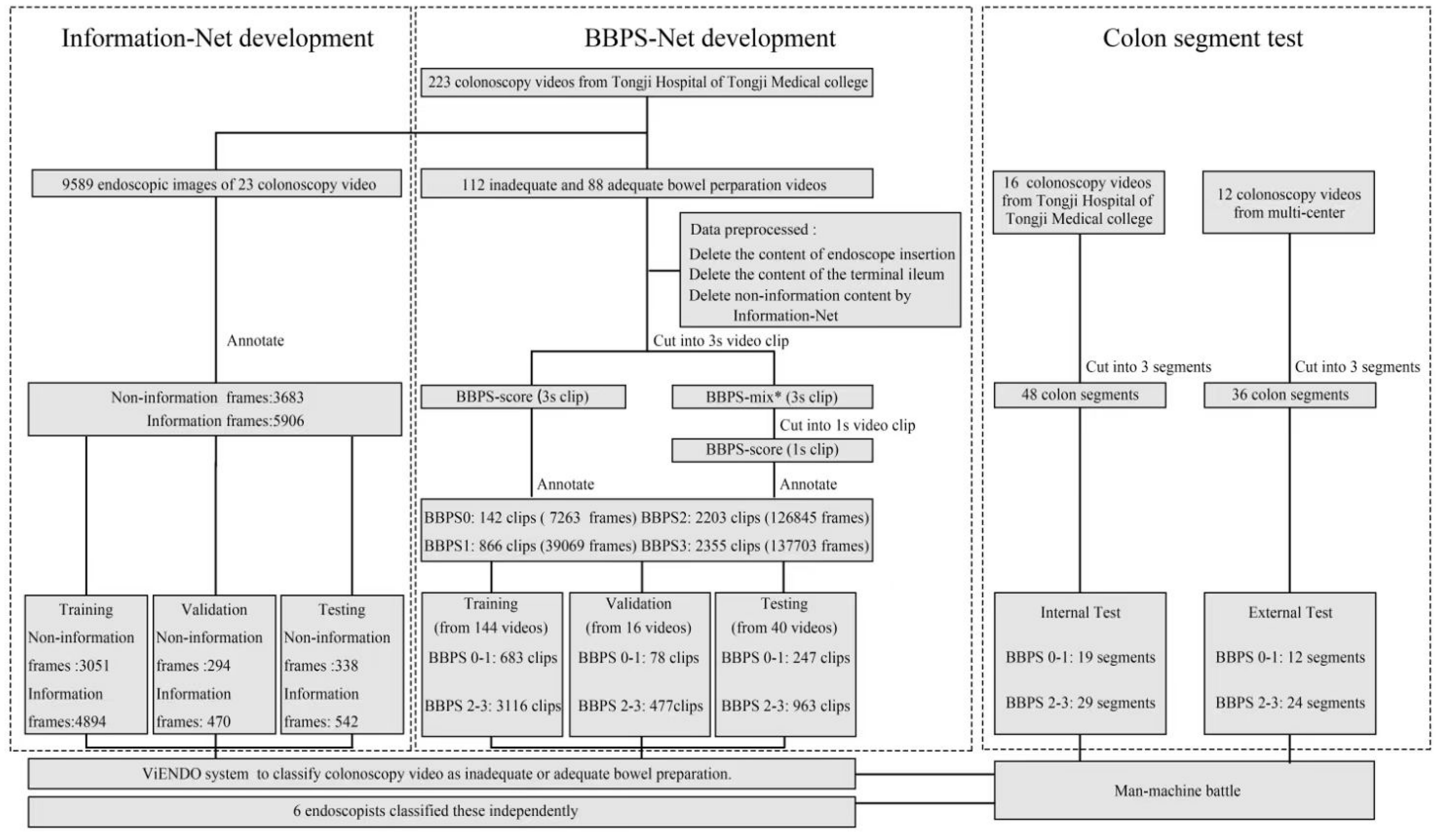
Overall study design. BBPS, Boston bowel preparation scale. ^*^BBPS-mix referred to the inclusion of different BBPS scores in a 3 s clip. For example, half of the clip was BBPS1, and the other half was BBPS2.

The overall study design is reported in Figure 1. A total of 223 colonoscopy videos were collected from our institution, and two CNNs were developed. First, 23 videos were used to develop Information-Net distinguishing between information and non-information frames. Information-Net could automatically filter out content that was unsuitable for BBPS scoring. Then, 200 videos (88 adequate and 112 inadequate) were used to develop BBPS-Net scoring bowel cleanliness. Besides normal diagnosis, these 200 videos also included a variety of clinical scenarios such as juvenile, IBD, unstable endoscope control, and withdrawal time of fewer than 6 min to ensure more generalizable and robust. A total of 10 endoscopists of varying seniority participated in the video recording. The colonoscopy equipment included CF-H290I, CF-260AI, and EC-760R-V/M. ViENDO was composed of Information-Net and BBPS-Net. The Institutional Ethics Board of Tongji Medical College, Huazhong University of Science and Technology (Wuhan, China) approved this study (TJ-IRB20230785). The informed consent was waived, as we only analyzed the de-identified data set.

### Development of Information-Net

Twenty-three videos were split into 9,859 frames and annotated by two endoscopists as 3,683 non-information and 5,906 information frames. The non-information included a variety of blurred scenes. After annotation, all frames were randomly divided into training (7,945 frames), validation (764 frames), and testing (880 frames) based on the ability to recognize ambiguity that we expect. Each frame was the black margin and resized to a standard resolution of 256 × 256 (32). After image normalization processing, Information-Net was trained based on ResNeXt101 architecture. A learning rate of 0.0001, a batch size of 64, and Adam’s optimizer were employed. Eventually, Information-Net classified each input frame as information and non-information frames with the maximum probability in the output. When applied to a real colonoscopy video, if five or more consecutive frames were identified as non-informational frames, these frames would not be rated by BBPS-Net.

### BBPS annotation

Two hundred videos were preprocessed as follows: (1) delete the content of endoscope insertion. (2) Delete the content of the terminal ileum. (3) Delete non-information content by Information-Net. (4) Cut into 3 s clips, which were used for annotation.

Before classification, our annotation group watched the 8 min BBPS instructional video and seriously studied the published definitions (12, 33). Next, two endoscopists labeled each 3 s clip with BBPS0, BBPS1, BBPS2, BBPS3, and BBPS-mix. Only when two endoscopists came to a consensus were the clips enrolled in the dataset. The entire annotation process was supervised and checked by an expert. BBPS0-1 and BBPS2-3 represented inadequate and adequate bowel preparation, respectively. BBPS-mix referred to the inclusion of different BBPS scores in a 3 s clip. For example, half of the clip was BBPS1, and the other half was BBPS2. BBPS-mix was further cut into 1 s clips and also annotated with BBPS scores. Finally, the 5,566 clips (310,880 frames) consisting of 142 BBPS0 clips (7,263 frames), 866 BBPS1 clips (39,069 frames), 2,203 BBPS2 clips (126,845 frames), and 2,355 BBPS3 clips (137,703 frames) were included.

### Development of BBPS-Net

**Figure 2:**
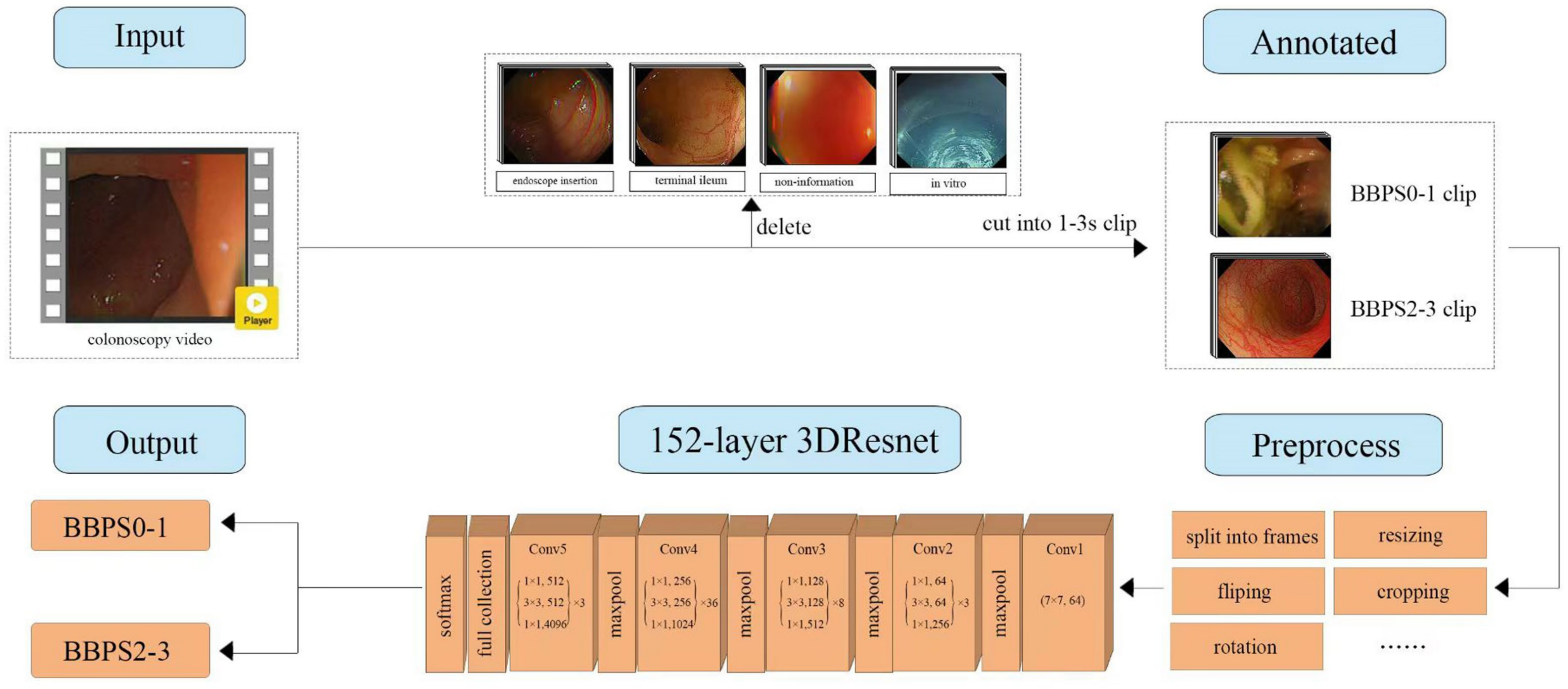
The workflow of the development of BBPS-Net. 3DResNet, three-dimensional residual network framework; BBPS, Boston bowel preparation scale.

The clips of 200 videos were randomly divided into training (144 videos), validation (16 videos), and testing (40 videos). The validation set was used to tune parameters and prevent overfitting. In the test set, the ratio of adequate to inadequate bowel preparation colonoscopy videos was 1:1. The workflow of the development of BBPS-Net is described in Figure 2. Before model training, each clip of the training and validation set was split into frames and then preprocessed by resizing, flipping, rotation, and cropping (32). Based on the 152-layer 3D residual network framework, the model can effectively extract the time series information to help the network better understand the video input. A learning rate of 0.0011, a batch size of 64, and a stochastic gradient descent optimizer were employed. The training was performed on two NVIDIA A40 GPUs. Eventually, BBPS-Net automatically classified each video clip as BBPS0-1 and BBPS2-3.

### Testing of ViENDO in colon segment test set

ViENDO consisted of Information-Net and BBPS-Net. We selected complete withdrawal colonoscopy videos from our and four other centers to further test ViENDO’s ability to predict BBPS segment scores in clinical scenarios. We manually divided the colon into three segments for each video by specifying the anatomical location. In our center, 48 colon segments of 16 patients were used as an internal segment test set. In the same way, 36 colon segments of 12 patients from Xiantao First People’s Hospital, Tianyou Hospital, Hubei Provincial Hospital of Traditional Chinese Medicine, and Yichang Central People’s Hospital were recorded as an external segment test set. To evaluate the accuracy difference between the algorithm and humans, 6 endoscopists and ViENDO classified colon segments as inadequate or adequate bowel preparation according to BBPS.

### Outcome measures and statistical analyses

The performance of Information-Net and BBPS-Net were evaluated with primary outcomes, including the area under the receiver operating characteristic curve (AUC), confusion matrix, accuracy, sensitivity, specificity, positive predictive value (PPV), negative predictive value (NPV), and *F*1-score. For the colon segment test dataset, the results of ViENDO’s performance and 6 endoscopists were evaluated with accuracy, sensitivity, and specificity in the internal and external datasets. Student *t*-test was used to compare accuracy differences between AI and the average of 6 endoscopists. Inter-observer agreement of 6 endoscopists was appraised using Fleiss’ kappa coefficient. All relevant data were analyzed using SPSS software (version 21.0).

## Results

### The performance of Information-Net

Information-Net reached the highest accuracy after 300 training epochs. In the test dataset, Information-Net discriminated the non-informational frames with an accuracy of 90.8%, sensitivity of 82.8%, specificity of 95.8%, PPV of 92.4%, NPV of 90.0%, and *F*1-score of 0.874 (Table 1).

**Table 1:** Performance of Information-Net and BBPS-Net.

	Accuracy	Sensitivity	Specificity	PPV	NPV	*F*1-score
Information-Net	90.80%	82.84%	95.76%	92.41%	89.95%	0.874
BBPS-Net	95.21%	84.21%	98.03%	91.63%	96.03%	0.878

PPV, positive predictive value; NPV, negative predictive value.

The corresponding confusion matrix is presented in Supplementary Figure S1.

### The performance of BBPS-Net

**Figure 3:**
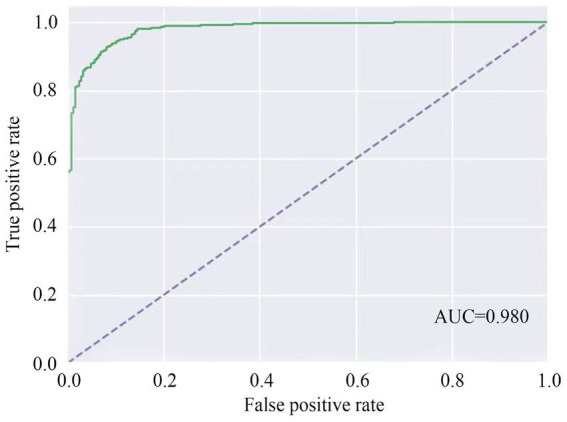
AUC of BBPS-Net to identify inadequate bowel preparation. AUC, area under the curve.

BBPS-Net reached the highest accuracy after 250 training epochs and generated an AUC of 0.98 to determine BBPS0-1 from video clips (Figure 3). The accuracy, sensitivity, and specificity of this model were 95.2%, 84.2%, and 98.0%, respectively (Table 1). The corresponding confusion matrix is demonstrated in Supplementary Figure S2.

### Analysis of misclassified video clips by BBPS-Net

**Figure 4:**
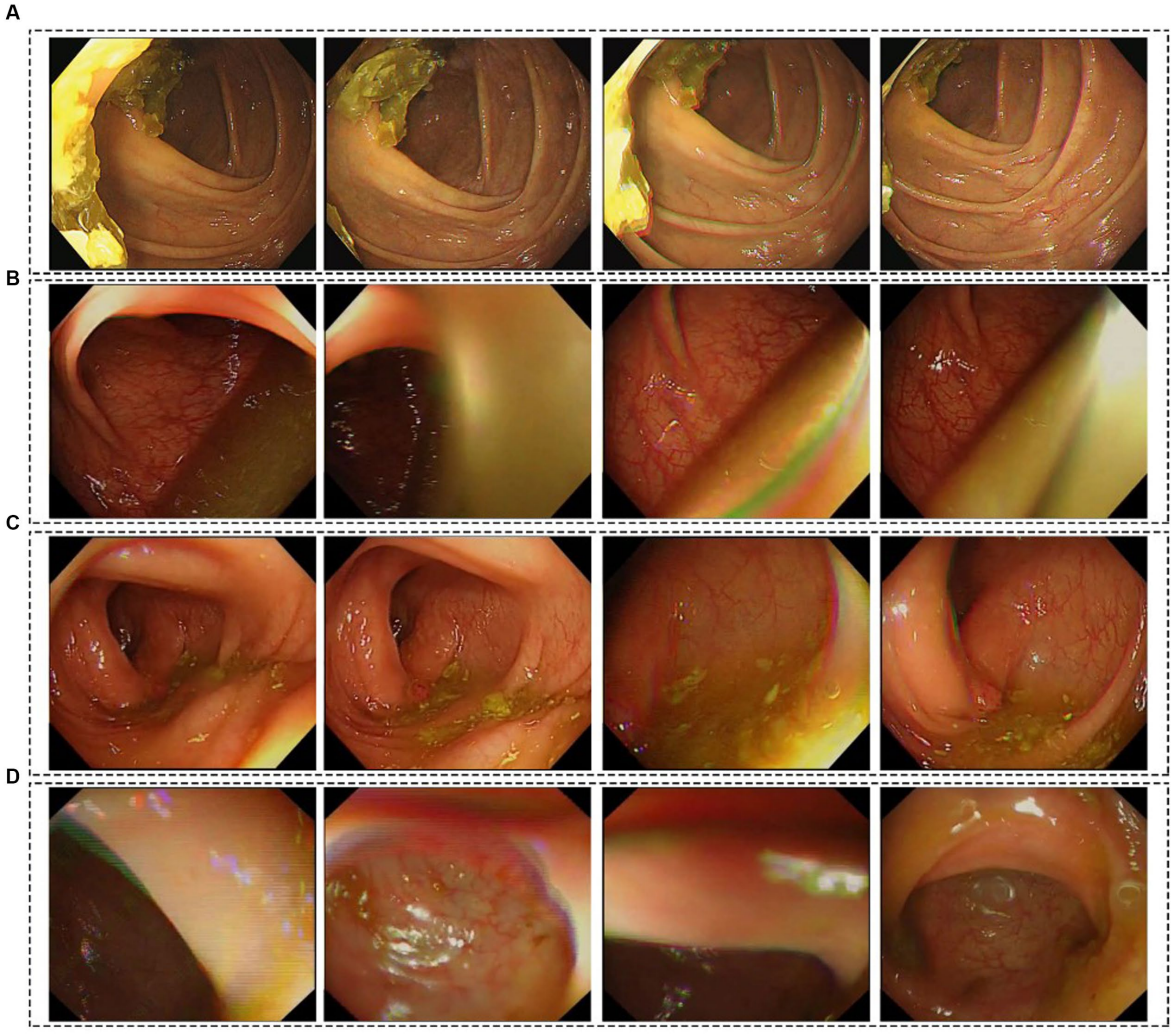
Illustration of misclassified video clips by BBPS-Net. **(A,B)** BBPS0-1 video clips were misclassified as BBPS2-3. **(C,D)** BBPS2-3 video clips were misclassified as BBPS0-1.

Figure 4 shows the specific misclassification of video clips by BBPS-Net. Two situations could cause BBPS-Net to misclassify BBPS0-1 as BBPS2-3, where only the edges of solid feces appeared in the endoscopic field of view or a large amount of liquid blocked the view of the endoscope, as shown in Figures 4A,B. Especially in the case of large amounts of liquids, we speculated that refracted light might cause BBPS-Net to be unable to distinguish whether a liquid is turbid or clear. Furthermore, BBPS-Net misclassified BBPS2-3 as BBPS0-1, mainly in scenes where feces were scattered or the picture changed rapidly, as presented in Figures 4C,D. Nevertheless, this kind of misclassification was relatively rare in our research.

### Comparison of performance between endoscopists and ViENDO in colon segment test set

**Figure 5:**
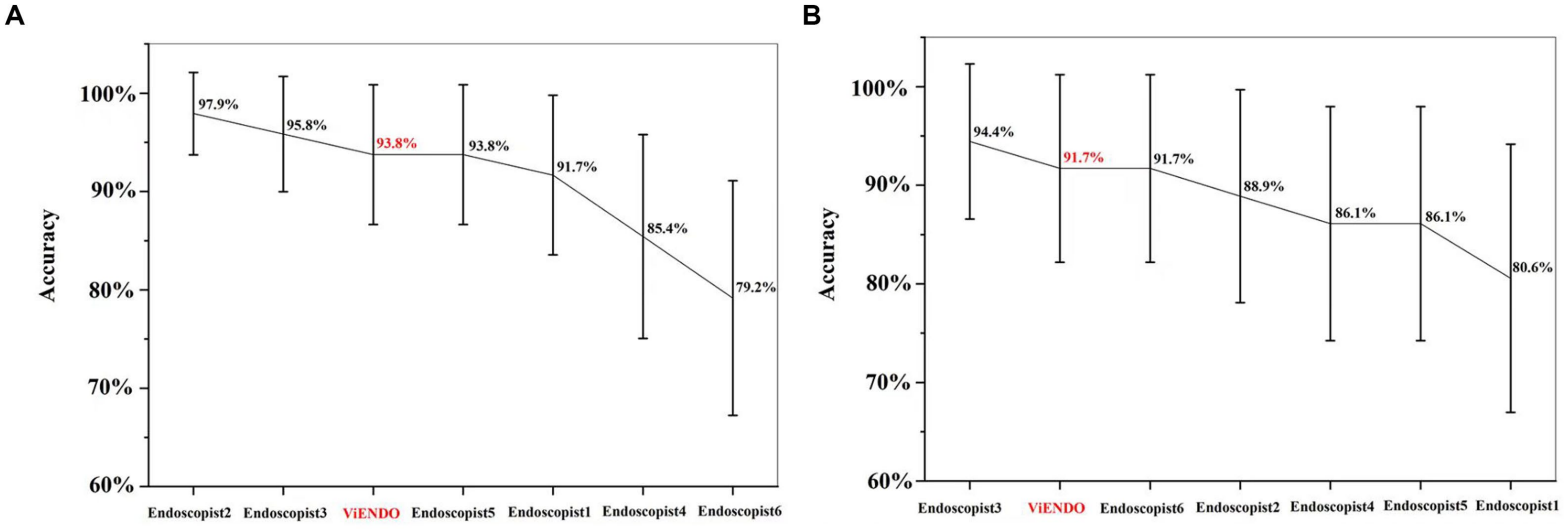
Accuracy of ViENDO and 6 endoscopists regarding their ability to assess bowel preparation with BBPS in colon segment test set. **(A)** Accuracy in the internal segment test dataset; **(B)** accuracy in the external segment test dataset.

In the internal segment test dataset, ViENDO detected inadequate bowel preparation with an accuracy of 93.8%, sensitivity of 84.2%, and specificity of 100%. In the external segment test dataset, ViENDO obtained an accuracy of 91.7%, sensitivity of 91.7%, and specificity of 91.7%. As shown in Figure 5, the accuracy of ViENDO in discriminating bowel preparation quality was slightly better compared to most endoscopists. Nevertheless, no statistically significant difference was found between the accuracy of AI and the average of 6 endoscopists (internal: 93.8% vs. 90.6%, *p* > 0.05; external: 91.7% vs. 88.0%, *p* > 0.05). The Fleiss’s kappa among 6 raters was 0.691 and 0.563 in the internal and external datasets (Supplementary Figure S3).

## Discussion

In this study, we developed a ViENDO system, including Information-Net and BBPS-Net, to assess the bowel preparation quality. Complete withdrawal colonoscopy videos from multiple centers were used to verify ViENDO’s performance, and we also conducted a human-machine contest.

The advance of AI in evaluating bowel preparation quality is a process that gradually gets closer to real-world clinical settings. In 2020, Su et al. (34) developed a CNN model trained with images to classify BBPS scores for bowel preparation, with an accuracy of 96.64% in distinguishing images. Nevertheless, the performance in colonoscopy videos was not discussed. At the same time, Zhou et al. (23) established an image-based ENDOANGEL system to distinguish inadequate from adequate bowel preparation and tested it in real colonoscopy videos. It took the worst BBPS frame to represent the entire video, obtaining an accuracy of 89.0%. Recently, Lee et al. (25) provided an AI-BBPS system trained with video frames closer to the clinical practice rather than report images. The AI-BBPS was validated in 10 s video clips with an accuracy of 85.3%, sensitivity of 81.0%, and specificity of 86.8%, respectively. As mentioned above, all published models were trained using images, and the accuracy in evaluating bowel cleanliness on colonoscopy videos did not exceed 90%. In our study, BBPS-Net, developed directly from videos, could identify insufficient bowel cleanliness from short video clips with an accuracy of 95.2% and AUC of 0.98. When applied to complete withdrawal colonoscopy videos, the accuracy of ViENDO in internal datasets was 93.8%. We also obtained external withdrawal colonoscopies from several other centers to demonstrate the model’s generalization, which still kept high accuracy. In addition, the short video dataset we used to train the model included not only normal diagnosis but also real clinical scenarios such as IBD, juveniles, unstable endoscope control, and withdrawal time of fewer than 6 min, which enhanced the robustness of the model. These clinical scenarios were not discussed in previous articles.

Unlike images, video clips contain a large number of spatiotemporal information during colonoscopy examination. AI trained directly with video through 3D-CNN is conducive to maintaining superior performance when applied to colonoscopy videos (26). This method has been applied in the field of polyp detection. Qadir et al. (30) achieved the best results (precision, 96.63%) on the CVC-ClinicVideoDB video dataset through bidirectional temporal information and the 3D-CNN model. Recently, González-Bueno Puyal et al. (31) provided a hybrid 2D/3D CNN architecture for polyp segmentation, which was validated on videos from the SUN polyp database and achieved higher performance (*F*1-score, 83.29%) and generalizability. To our knowledge, we are the first to apply 3D-CNN technology to evaluate bowel preparation and achieve good performance in real colonoscopy videos.

In addition, the previous AI system was a modified scoring method different from traditional standard BBPS (24), which has not been validated by other studies, and its widespread use is limited. We used the highly validated standard BBPS as the endpoint. ViENDO could predict three colon segments separately as inadequate or adequate bowel preparation and has the potential for universal application.

This study had a few limitations. Firstly, for traditional BBPS, recognizing the anatomical location to separate the colon into three segments is a prerequisite for scoring, but this remains a challenge for AI to date. AI recognition of colon anatomical location is also important for other colon diseases and should be addressed in the future. We manually segment the colon in our work. Secondly, tetrataxonomy was not studied in the present research, but the binary classification of bowel preparation is sufficient to provide follow-up recommendations for subjects undergoing colonoscopy in clinical practice (5, 35). Thirdly, ViENDO has not yet been applied in clinical practice. For better application, we will further accelerate the model and deploy it in a hardware box. If the hardware box is already available in the endoscopy center, that latter is a low-cost thing to do. Fourthly, we only confirmed the clinical feasibility of ViENDO in retrospectively collected colonoscopy videos. Further prospective clinical studies should be carried out to verify the efficiency of this system in real-time application.

To conclude, we proposed a highly precise and reliable 3D-CNN-based AI instrument to evaluate full-length bowel preparation according to traditional BBPS. Further real-time verification of this tool might help optimize bowel cleansing, provide the valuable gift of time to physicians, and improve the quality of colonoscopy reports.

## Data availability statememt

The original contributions presented in the study are included in the article/Supplementary material, further inquiries can be directed to the corresponding authors.

## Ethics statement

The studies involving humans were approved by The Ethical Committees of Tongji Hospital of Tongji Medical College have approved this study (TJ-IRB20230785). The studies were conducted in accordance with the local legislation and institutional requirements. The ethics committee/institutional review board waived the requirement of written informed consent for participation from the participants or the participants’ legal guardians/next of kin because we only analyzed de-identified data, the informed consent was waived.

## Author contributions

LF: Visualization, Writing – original draft, Writing – review & editing. JX: Visualization, Writing – original draft. XJ: Methodology, Visualization, Writing – original draft. LC: Data curation, Formal analysis, Writing – original draft. ShX: Data curation, Formal analysis, Writing – original draft. BL: Methodology, Visualization, Writing – review & editing. JH: Data curation, Formal analysis, Methodology, Writing – original draft. KZ: Data curation, Formal analysis, Writing – original draft. JuL: Methodology, Visualization, Writing – original draft. SuX: Data curation, Formal analysis, Methodology, Writing – original draft. JG: Data curation, Formal analysis, Methodology, Writing – original draft. CY: Methodology, Visualization, Writing – original draft. QT: Resources, Writing – original draft. HL: Resources, Writing – original draft. JZ: Resources, Writing – original draft. RC: Resources, Writing – original draft. DT: Writing – review & editing. XL: Writing – review & editing. FX: Conceptualization, Writing – review & editing. JiL: Conceptualization, Writing – review & editing.
