# Supplementary material for: Development and validation of a three-dimensional deep learning-based system for assessing bowel preparation on colonoscopy video
Source: Front Med (Lausanne). 2023 Dec 18;10:1296249. doi: 10.3389/fmed.2023.1296249 (PMC10757977; doi:10.3389/fmed.2023.1296249)
Supplement: Supplementary file 1 [file Data_Sheet_1.docx]

***Supplementary Material***

**Supplementary Figures**


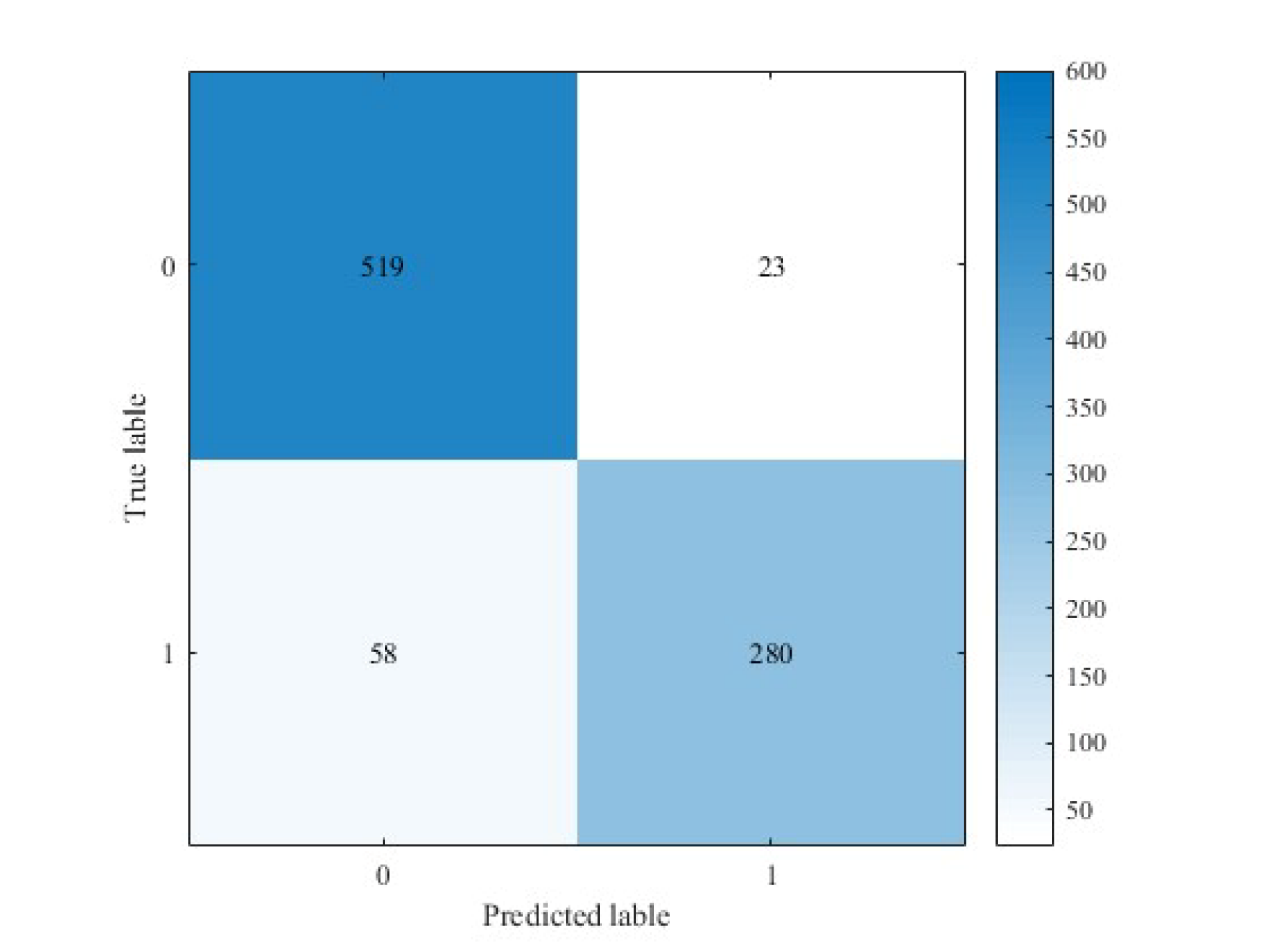
**Supplementary Figure 1** Confusion matrix for Information-Net.


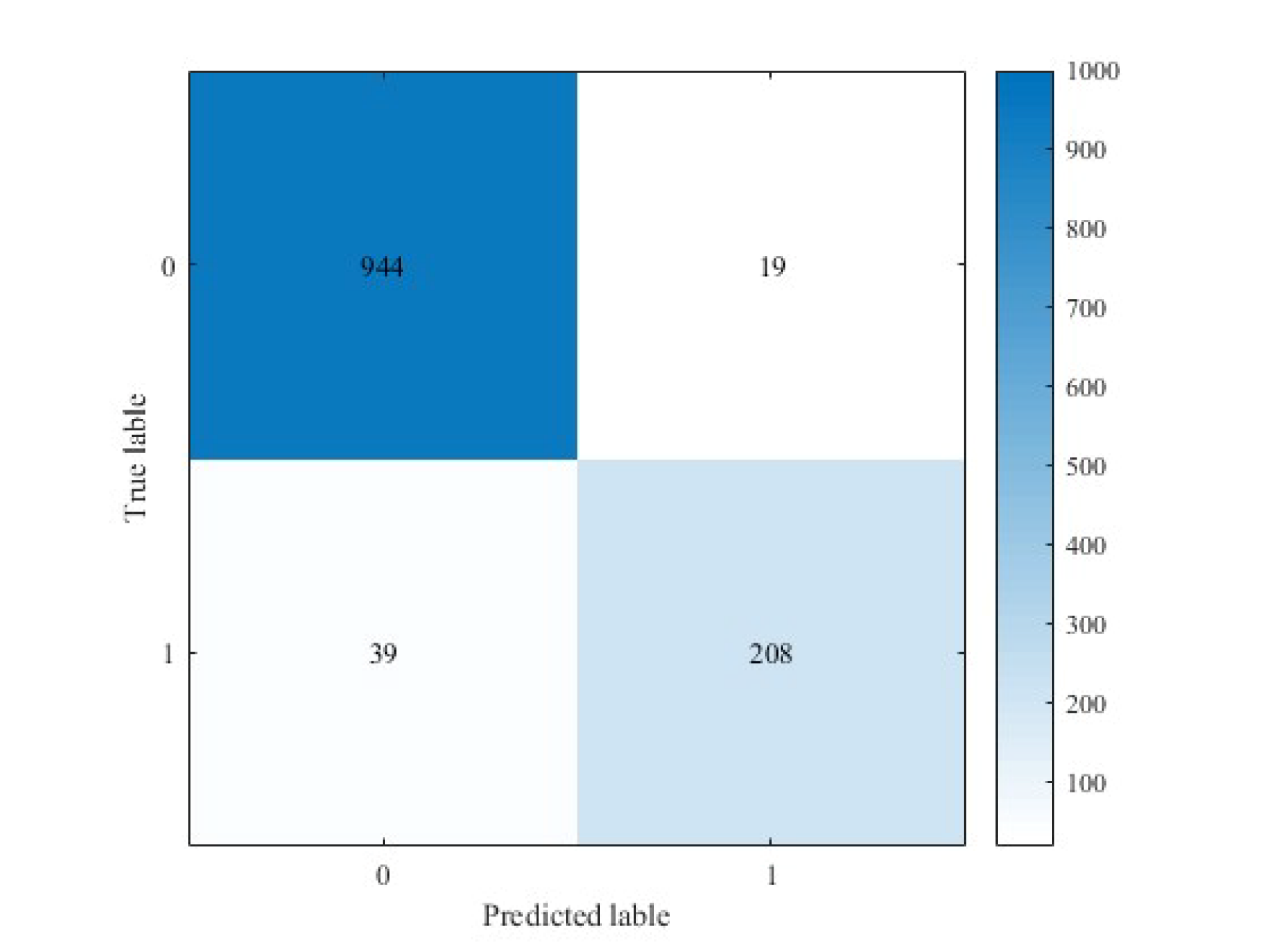


**Supplementary Figure 2** Confusion matrix for BBPS-Net.


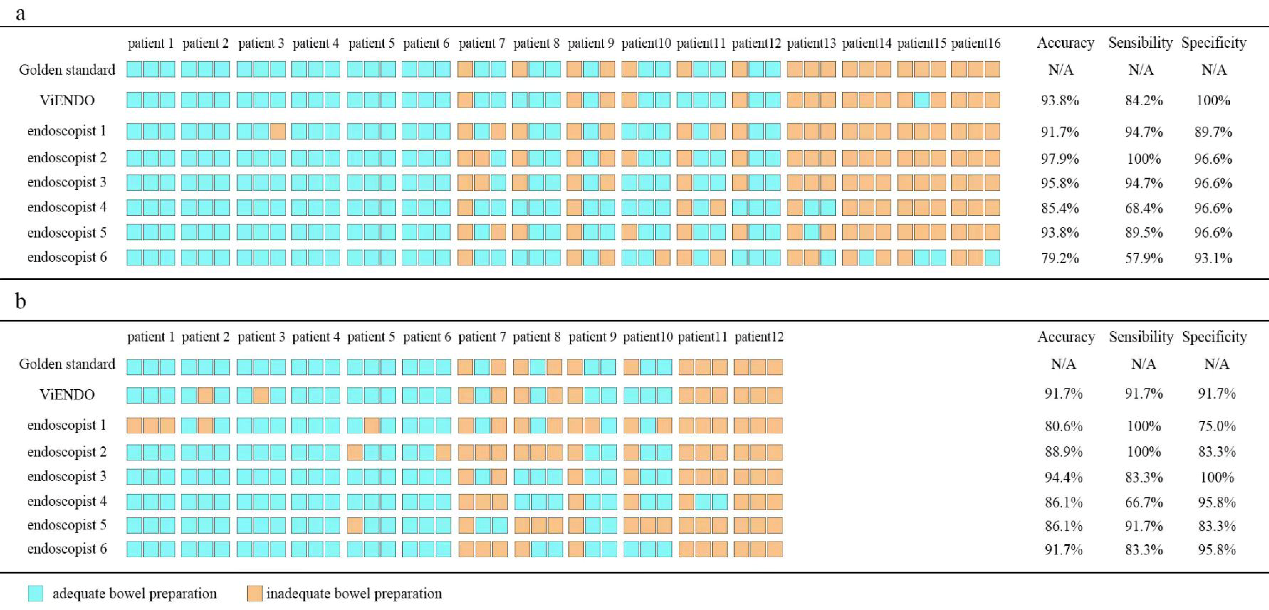


**Supplementary Figure 3** Evaluation of the bowel preparation quality by ViENDO and 6 endoscopists in the colon segment video dataset. Each small grid represented a bowel segment.
